# Supplementary material for: Randomized Crossover Clinical Trial of Nicotinamide Riboside and Coenzyme Q10 on Metabolic Health and Mitochondrial Bioenergetics in CKD
Source: medRxiv. 2024 Aug 23:2024.08.23.24312501. Preprint. [Version 1] doi: 10.1101/2024.08.23.24312501 (PMC11370499; doi:10.1101/2024.08.23.24312501)

medRxiv preprint doi: https://doi.org/10.1101/2024.08.23.24312501; this version posted August 23, 2024. The copyright holder for this preprint
(which was not certified by peer review) is the author/funder, who has granted medRxiv a license to display the preprint in perpetuity.
It is made available under a CC-BY 4.0 International license .

Supplemental Figure 1. CONSORT diagram illustrating data completeness of the various outcomes in the Cross-over Randomized Controlled Trial of Coenzyme Q10 and Nicotinamide Riboside in CKD (CoNR Trial).

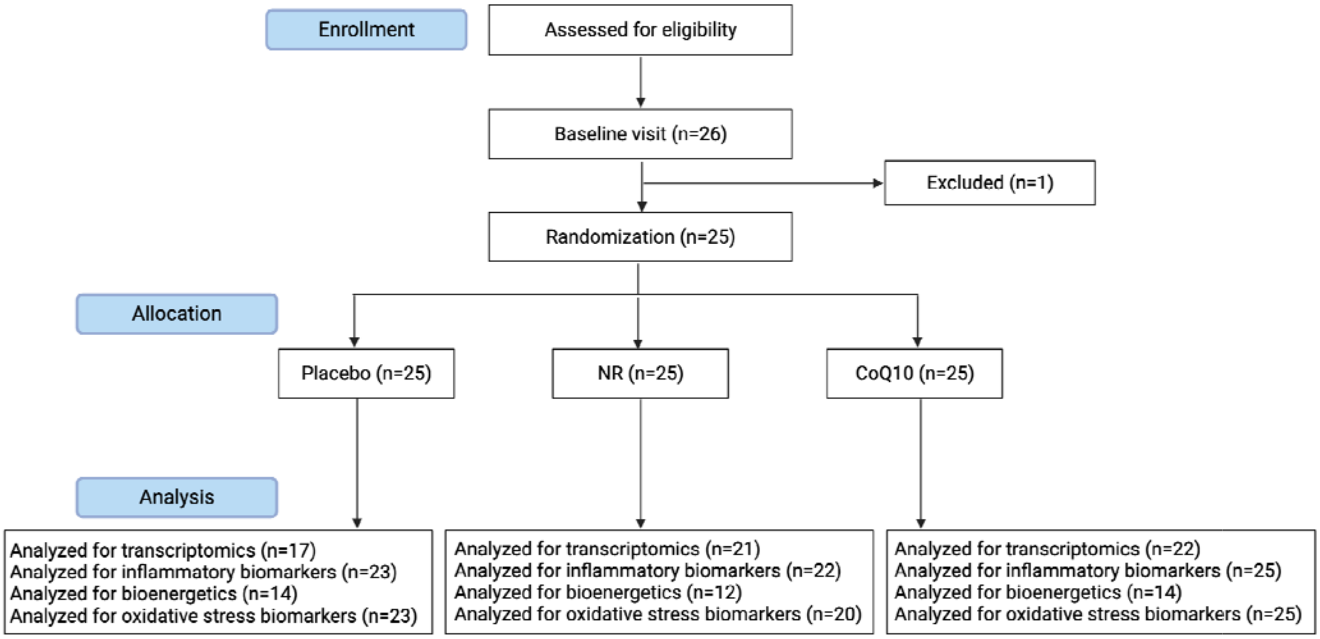

medRxiv preprint doi: https://doi.org/10.1101/2024.08.23.24312501; this version posted August 23, 2024. The copyright holder for this preprint (which was not certified by peer review) is the author/funder, who has granted medRxiv a license to display the preprint in perpetuity.
It is made available under a CC-BY 4.0 International license .

Supplemental Figure 2. Study design and sample size of the Cross-over Randomized Controlled Trial of Coenzyme Q10 and Nicotinamide Riboside in CKD (CoNR Trial).

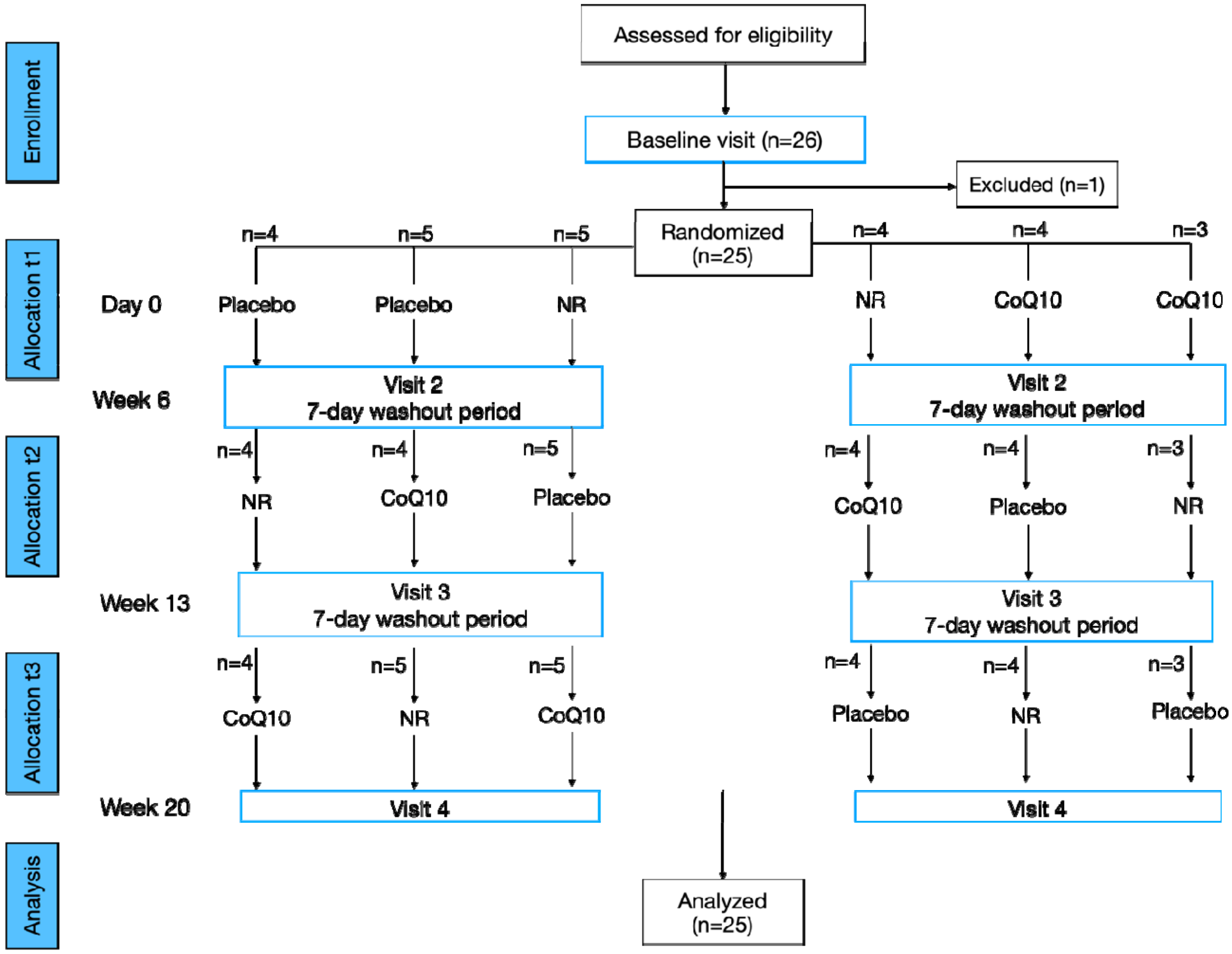

medRxiv preprint doi: https://doi.org/10.1101/2024.08.23.24312501; this version posted August 23, 2024. The copyright holder for this preprint (which was not certified by peer review) is the author/funder, who has granted medRxiv a license to display the preprint in perpetuity.
It is made available under a CC-BY 4.0 International license .

Supplemental Figure 3. Gene ontology analysis representing altered molecular functions (mf) in whole blood (A) post NR and B) CoQ10 supplementation. Bars represent the P-value (–Log10). Top 20 terms are shown.

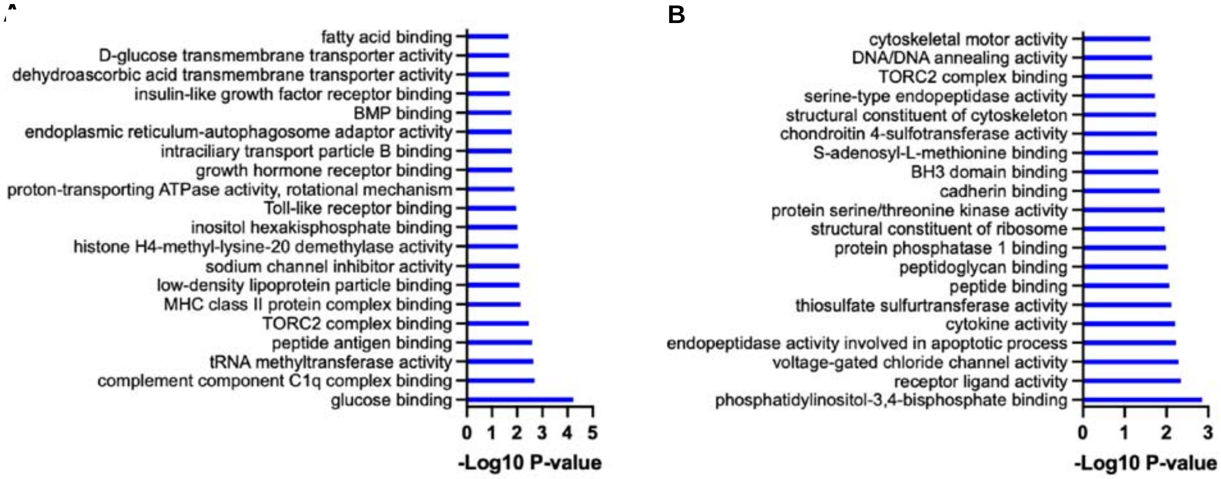

medRxiv preprint doi: https://doi.org/10.1101/2024.08.23.24312501; this version posted August 23, 2024. The copyright holder for this preprint (which was not certified by peer review) is the author/funder, who has granted medRxiv a license to display the preprint in perpetuity.
It is made available under a CC-BY 4.0 International license .

Supplemental Table 1. Sub-population characteristics of participants with monocyte and lymphocyte bioenergetics (n=14) compared to the entire cohort (n=25).

**Baseline characteristics**	N=14	N=25
Age (years), mean (SD)	58.7 (12.7)	61.0 (11.6)
Male, n (%)	7 (50)	15 (60.0)
Race, n (%)		
Asian	1 (7)	3 (12.0)
Black/African-American	1 (7)	1 (4.0)
Hispanic	0 (0)	1 (4.0)
Native Hawaiian/Pacific Islander	0 (0)	1 (4.0)
White	12 (85)	20 (80.0)
BMI (kg/m^2), mean (SD)	26.7 (4.6)	27.7 (5.2)
SBP (mmHg), mean (SD)	130.7 (27)	129 (22)
eGFR (mL/min/1.73m^2), mean (SD)	36.2 (7.2)	36.9 (9.2)
Serum triglyceride (mg/dL), mean (SD)	162 (98)	166 (107)
Serum HDL (mg/dL), mean (SD)	57 (17)	58 (17)
Serum LDL (mg/dL), mean (SD)	107 (38)	94 (33)
Physical activity past month (hours), median (IQR)	28 (0.3, 35)	13 (2, 30)
Six-minute walking distance (meters), mean (SD)	432 (66)	414 (69)
VO_2 peak (mL/kg/min), mean (SD)	22 (4.2)	21 (4.5)
ADL score, mean (SD)	8.0 (0)	8.0 (0.2)
Diabetes, n (%)	2 (14.0)	4 (16.0)
Current smoker, n (%)	2 (14.0)	3 (12.0)
ACE/ARB, n (%)	11 (78)	18 (72.0)
Statins, n (%)	7 (50)	14 (56.0)
Erythropoietin, n (%)	0 (0)	1 (4.0)

Abbreviations: Chronic kidney disease was defined as estimated glomerular filtration rate <60 ml/min per m^2. SD, standard deviation; IQR, interquartile range; SBP, systolic blood pressure; eGFR, estimated

medRxiv preprint doi: https://doi.org/10.1101/2024.08.23.24312501; this version posted August 23, 2024. The copyright holder for this preprint (which was not certified by peer review) is the author/funder, who has granted medRxiv a license to display the preprint in perpetuity.
It is made available under a CC-BY 4.0 International license .

glomerular filtration rate; ADL, Activities of Daily Living, ACE/ARB, angiotensin converting enzyme inhibitors/ angiotensin-receptor blockers.

Supplemental Table 2. Summary of secondary outcome of plasma inflammatory biomarkers comparing NR and CoQ10 with placebo. Linear mixed effects modeling was used to estimate treatment effects compared to placebo. Unadjusted P-values, **effect sizes**, and 95% CIs are shown. The comparisons that withstood multiple comparison adjustment are shown in bold.

Endpoint	NR vs placebo Effect size (95% CI)	NR vs placebo P-value	CoQ10 vs placebo Effect size (95% CI)	CoQ10 vs placebo P-value
sCRP, mg/dL	-0.32 (-0.91 to 0.29)	0.13	-0.45 (-1.05 to -0.17)	0.04
IL-6, pg/mL	0.14 (-0.44 to 0.73)	0.48	-0.11 (-0.67 to 0.46)	0.52
IL-10, pg/mL	0.23 (-0.36 to 0.81)	0.10	-0.1 (-0.67 to 0.46)	0.57
IL-12, pg/mL	0.2 (-0.39 to 0.78)	0.07	0.03 (-0.53 to 0.6)	0.61
IL-13, pg/mL	-0.35 (-0.97 to 0.27)	0.05	-0.37 (-0.1 to -0.25)	**0.02**
IL-2, pg/mL	0.65 (0.04 to 1.24)	**<0.01**	0.25 (-0.33 to 0.81)	0.17
IL-4, pg/mL	-0.13 (-0.71 to 0.46)	0.41	-0.37 (-0.94 to 0.02)	0.16
IL-8, pg/mL	0.06 (-0.51 to 0.63)	0.72	0.01 (-0.56 to 0.57)	0.97
TNF-α, pg/mL	0.26 (-0.32 to 0.85)	0.13	0.01 (-0.56 to 0.57)	0.95
IFN-γ, pg/mL	0.55 (-0.06 to 1.15)	0.12	0.06 (-0.51 to 0.62)	0.80

medRxiv preprint doi: https://doi.org/10.1101/2024.08.23.24312501; this version posted August 23, 2024. The copyright holder for this preprint (which was not certified by peer review) is the author/funder, who has granted medRxiv a license to display the preprint in perpetuity.
It is made available under a CC-BY 4.0 International license .

Supplemental Table 3. Summary of secondary outcome of plasma inflammatory biomarkers comparing NR and CoQ10 with placebo. Linear mixed effects modeling was used to estimate treatment effects compared to placebo. Unadjusted P-values, mean differences, and 95% CI are shown. The comparisons that withstood multiple comparison adjustment are shown in bold.

Endpoint	Mean placebo (SD)	NR vs placebo (95% CI)	NR vs placebo P-value	CoQ10 vs placebo (95% CI)	CoQ10 vs placeb P-value
F2 Isoprostanes, pg/mL	1.26 (0.41)	0.28 (0.00 to 0.25)	0.05	-0.02 to 0.23	0.09
5 series F2 Isoprostanes, pg/mL	1.1 (0.4)	-0.16 (-0.28 to -0.03)	**0.02**	-0.11 (-0.22 to 0.00)	**0.04**
5-F2t-IsoP, pg/mL	0.27 (0.12)	-0.04 (-0.07 to -0.01)	**<0.01**	-0.03 (-0.06 to 0.00)	**0.03**
5-F2c-IsoP, pg/mL	0.86 (0.31)	-0.09 (-0.18 to 0.02)	0.06	-0.07 (-0.16 to 0.03)	0.17
15-F2t-IsoP, pg/mL	0.15 (0.05)	0.00 (-0.02 to 0.02)	0.78	0.00 (-0.02 to 0.02)	0.96

medRxiv preprint doi: https://doi.org/10.1101/2024.08.23.24312501; this version posted August 23, 2024. The copyright holder for this preprint (which was not certified by peer review) is the author/funder, who has granted medRxiv a license to display the preprint in perpetuity.
It is made available under a CC-BY 4.0 International license .

Supplemental Figure 4. Oxygen consumption rates of A) monocytes and C) T-cells and extracellular acidification rates (ECAR) of B) monocytes and D) T-cells measured by an XFe24 Seahorse Analyzer (n=14). The *Cell Mito Stress* test involved an injection of 1µM oligomycin (OMY), followed by an injection of 0.6-1.0 µM carbonyl cyanide-4-(trifluoromethoxy)phenylhydrazone (FCCP), and an injection of 10µM antimycin A (AMA) in a XFe24 Analyzer. Data are normalized to cell number per well. Data expressed as mean±SEM.

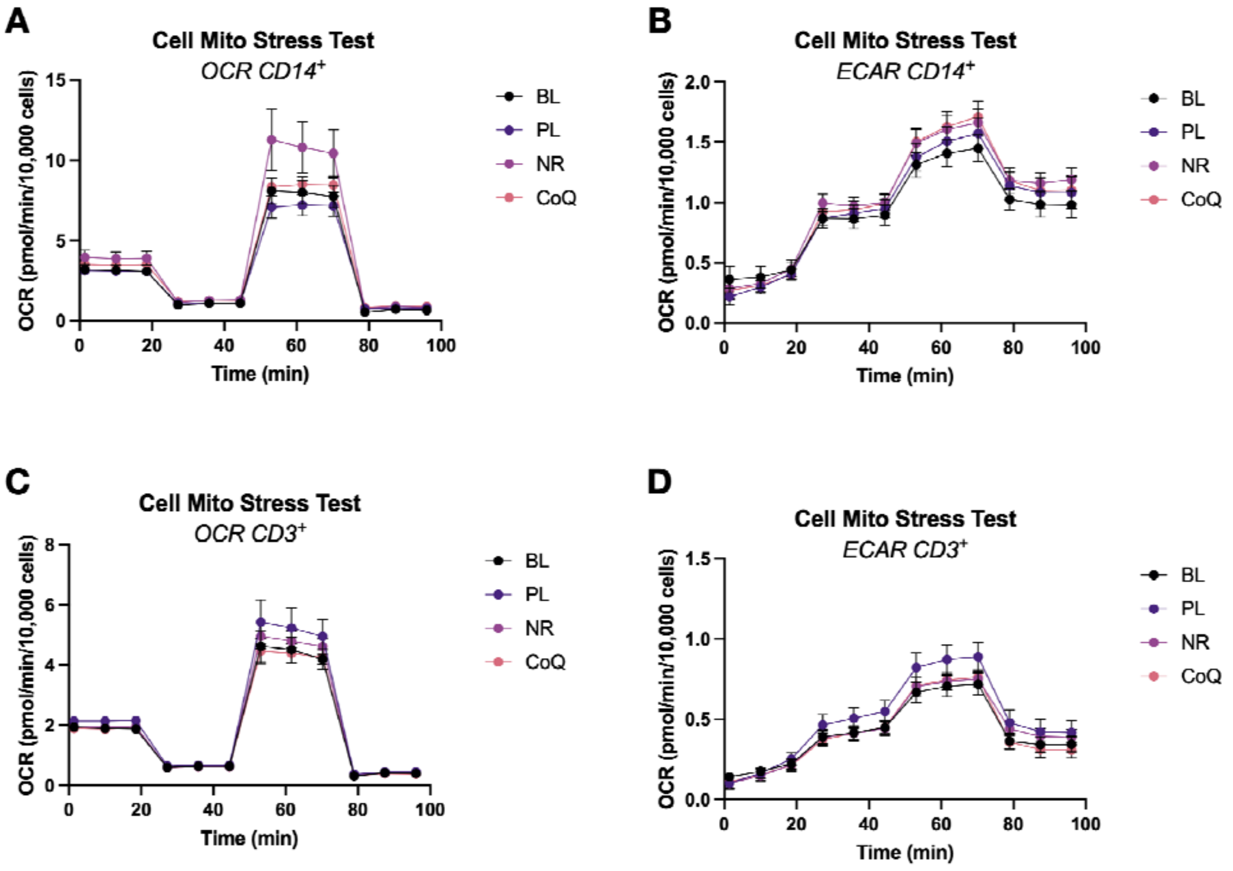

medRxiv preprint doi: https://doi.org/10.1101/2024.08.23.24312501; this version posted August 23, 2024. The copyright holder for this preprint (which was not certified by peer review) is the author/funder, who has granted medRxiv a license to display the preprint in perpetuity.
It is made available under a CC-BY 4.0 International license .

Supplemental Figure 5. The effects of nicotinamide riboside (NR), and coenzyme Q 10 (CoQ10) on T-cell (CD3$^+$) bioenergetics (n=14). Bioenergetic parameters include A) basal respiration, B) proton leak respiration, C) maximal respiratory capacity E) spare respiratory capacity, E) ATP-linked respiration, F) bioenergetic health index , calculated by the log [(ATP-linked respiration x spare respiratory capacity)/(proton leak x non-mitochondrial respiration]. The box plots represent median and IQR and the whiskers represent minimum and maximum values. *P<0.05 compared to placebo.

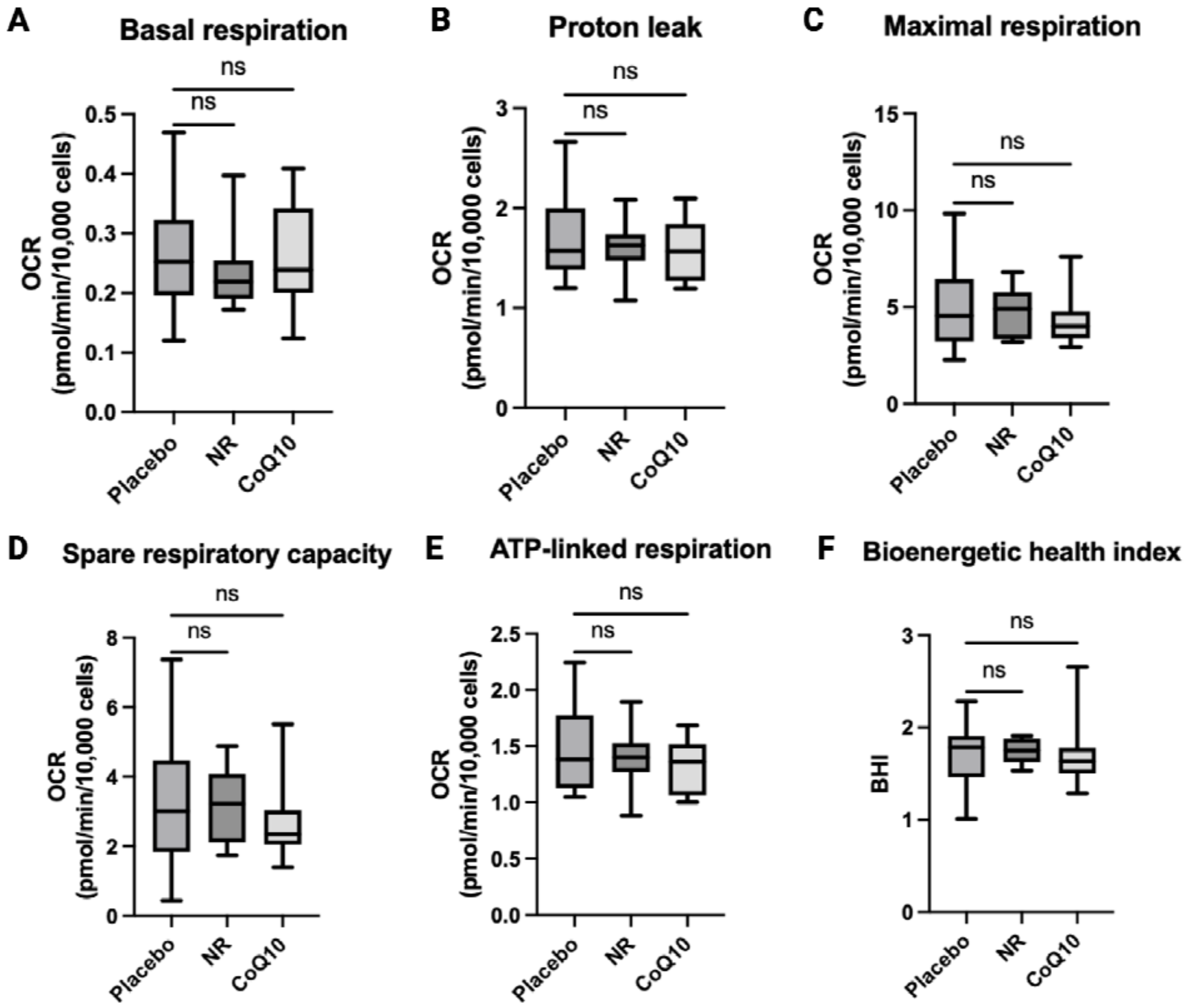

Supplement: Supplement 1 [file NIHPP2024.08.23.24312501v1-supplement-1.pdf]
